# Biochar Derived from Agro-Industrial Coconut Shell Waste for the Removal of Aflatoxin B1 Using an In Vitro Model That Considers Buffer Solutions and an In Vitro Avian Digestion Model

**DOI:** 10.3390/foods15071165

**Published:** 2026-03-30

**Authors:** Karla S. García-Salazar, Raquel López-Arellano, Jesús A. Maguey-Gonzalez, Juan D. Latorre, Elvia Adriana Morales Hipólito, Maykel González-Torres, Jorge L. Mejía-Méndez, Alma Vázquez-Durán, Guillermo Tellez-Isaias, Abraham Méndez-Albores, Bruno Solis-Cruz, Daniel Hernandez-Patlan

**Affiliations:** 1 Nanotechnology Engineering Division, Polytechnic University of the Valley of Mexico, Tultitlan 54910, Mexico; karla.garcia.salazar@upvm.edu.mx; 2 Laboratory 5: Laboratorio de Ensayos de Desarrollo Farmacéutico (LEDEFAR), Multidisciplinary Research Unit, Superior Studies Faculty at Cuautitlan (FESC), National Autonomous University of Mexico (UNAM), Cuautitlan Izcalli 54714, Mexico; lopezar@unam.mx (R.L.-A.); eadriana_mh@comunidad.unam.mx (E.A.M.H.); 3 Division of Agriculture, Department of Poultry Science, University of Arkansas, Fayetteville, AR 72701, USA; jm201@uark.edu (J.A.M.-G.); jl115@uark.edu (J.D.L.); 4 Departamento de Ciencias Químicas, Superior Studies Faculty at Cuautitlan (FESC), National Autonomous University of Mexico (UNAM), Cuautitlán Izcalli 54714, Mexico; maykel.gonzalez@unam.mx; 5 Escuela de Ingeniería y Ciencias, Tecnologico de Monterrey, Epigmenio González 500, San Pablo 76130, Mexico; mejia.jorge@tec.mx; 6 Unidad de Investigación Multidisciplinaria L14 (Alimentos, Micotoxinas, y Micotoxicosis), Facultad de Estudios Superiores Cuautitlán (FESC), Universidad Nacional Autónoma de México (UNAM), Cuautitlán Izcalli 54714, Mexico; almavazquez@comunidad.unam.mx (A.V.-D.); albores@unam.mx (A.M.-A.); 7 Gut Healt LLC, Fayetteville, AR 72703, USA; gtellez@uark.edu

**Keywords:** biochar, coconut shell, agro-industrial waste, aflatoxin B1, adsorption capacity, in vitro avian digestion model

## Abstract

The use of agro-industrial waste to obtain biochar has emerged as an environmentally friendly, low-cost, effective, profitable, and sustainable strategy for the removal of aflatoxin B1 (AFB1), a highly toxic and carcinogenic mycotoxin of importance in poultry production systems because it can cause serious economic losses, affect hatchability, egg production, and the growth of birds, and can cause their death. In this sense, the objective of the present study was to obtain a sustainable and low-cost biochar derived from agro-industrial coconut shell waste (BCS) and evaluate its AFB1 adsorption capacity using a conventional method based on buffer solutions and an in vitro avian digestion model that simulates the conditions of the gastrointestinal tract of the broiler chicken. The results showed that the adsorption capacity of BCS on AFB1 (250 ng/mL) at both pH 5.0 and 1.2 was close to 100%, while at pH 6.8, the adsorption of AFB1 was 86.24%. However, in the in vitro avian digestibility model, the adsorption capacity of BSC on AFB1 was 32.96%, thus highlighting the importance of considering factors that can affect the adsorption capacity of materials before in vivo studies, as this can lead to overestimations of results and, therefore, ineffective treatments or unexpected results in animals.

## 1. Introduction

Aflatoxin B1 (AFB1) is a secondary metabolite produced mainly by the filamentous fungi *Aspergillus flavus* and *Aspergillus parasiticus* when relative humidity conditions are high (greater than 80%), and the temperature ranges between 25 and 35 °C during the storage of grains, cereals, and seeds [1,2]. This mycotoxin is highly toxic and carcinogenic, causing serious economic losses in poultry production systems as it affects hatchability, growth, meat and egg production, and can even cause death, given the susceptibility of birds to infectious diseases [3], due to effects such as hepatotoxicity, immunosuppression, intestinal barrier dysfunction, dysbiosis, and oxidative stress [1].

In this regard, various strategies have been proposed to mitigate the problems associated with AFB1, such as radiation, ozonation, or chemical inactivation. However, due to the excellent stability of AFB1 at high temperatures, it is difficult to eliminate this mycotoxin using conventional feed processing methods. Furthermore, the methods that have shown promising results are costly and have the disadvantage of potentially reducing the nutritional value of food, as well as promoting the formation of toxic byproducts [4], and in the specific case of chemical methods, these can also affect the palatability of feed [5]. In the case of biological methods for mitigating AFB1 in poultry, such as the use of bacteria, yeasts, or enzymes, this is considered a promising and safe approach, but they have the disadvantage that their production costs are higher than implementation costs compared to traditional physical or chemical methods [6]. Therefore, one of the most practical and important decontamination strategies in poultry farming has been the use of adsorbent materials, which are added to feed to bind AFB1 through the gastrointestinal tract [7]. The most frequently used adsorbents in poultry farming have been inorganic compounds, such as zeolites, aluminosilicates, and clays. Nonetheless, these adsorbents are nonspecific, leading to feed efficiency problems, and many can release toxic components such as heavy metals and dioxins [8].

Considering this background, in recent years, the use of agro-industrial waste to obtain biochar has emerged as an environmentally friendly, low-cost, effective, profitable, and sustainable strategy for contaminant removal, given its adsorbent properties arising from its high surface area, porosity, and presence of functional groups on the surface [9,10]. In fact, it is estimated that of the 1300–2100 million tons of agro-industrial wastes generated annually in the world, coconut shell contributes 2.4–3.8% (50 million tons), positioning it as one of the most frequently generated agro-industrial waste products generated by the agricultural industry [11]. Thus, to take advantage of these agro-industrial wastes, the development of materials such as biochars has been pursued for their potential applications as adsorbents for the removal of contaminants of organic origin, microplastics, heavy metals, and even nutrients from wastewater and feed, since, by their own nature, they do not have good adsorbent properties [12,13].

Therefore, the objective of the present study was to obtain a sustainable and low-cost biochar derived from agro-industrial coconut shell waste (BCS) and evaluate its potential AFB1 adsorbtion capacity in an in vitro avian digestion model that simulates the conditions of the gastrointestinal tract of the broiler chicken in terms of the presence of feed, pH, enzymatic activity, and residence time in the crop, proventriculus, and an intestinal section to better understand its behavior, since most adsorption studies are carried out in aqueous solutions, which can result in overestimations and variability of results in the animals.

## 2. Materials and Methods

### 2.1. Chemicals and Reagents

Aflatoxin B1 (AFB1, purity ≥ 98%) was purchased from Cayman Chemical Company (Ann Arbor, MI, USA), while dimethyl sulfoxide (DMSO, purity ≥ 99.5%, analytical grade) was purchased from Sigma-Aldrich (Saint Louis, MO, USA). Acetonitrile (ACN, HPLC grade, purity ≥ 99.9%), methanol (MeOH, HPLC grade, purity ≥ 99.9%), hydrochloric acid (HCl, reagent grade, purity 36.5–38.0%), sodium hydroxide (NaOH, reagent grade, purity ≥ 98.0%), and sodium bicarbonate (NaHCO_3_, reagent grade, purity 99.7–100.3%) were purchased from JT Baker (Radnor, PA, USA). Pepsin (1:10,000) and pancreatin (8×) were obtained from Bio Basic (Markham, ON, Canada). Water was purified using a Milli-Q system (Merck-Millipore, Darmstadt, Germany).

### 2.2. Preparation of BCS

The coconut shells used to obtain the biochar were obtained from fruits harvested in San Luis Acatlán, Guerrero, Mexico. These agro-industrial waste products were then carefully washed with distilled water to remove surface impurities and organic matter and were left to dry at room temperature for two weeks to remove moisture. Subsequently, these agro-industrial residues were subjected to a controlled carbonization process in a muffle furnace (Marla J-15, MARLA Equipos, Nezahualcóyotl, Mexico) at 400 °C for 60 min in the presence of oxygen to remove volatile compounds and promote the formation of initial carbonaceous structures with an incipient pore network.

After carbonization, the resulting material was removed from the muffle furnace and cooled to room temperature. The carbonized material was then ground in a mortar to obtain a fine powder to reduce the particle size. The activation stage consisted of subjecting the material to a second heating at 720 °C for 60 min to develop the porous structure and generate active adsorption sites. Finally, the samples were cooled to room temperature and sieved through a No. 35 mesh (500 μm) to homogenize the particle size.

### 2.3. Characterization of the Biochar

#### 2.3.1. Particle Size Determination by Laser Diffraction

The particle size of BCS was determined using a laser diffraction analyzer equipped with a Tornado dry powder system (LS 13 320, Beckman Coulter, Miami, FL, USA). This particle size analyzer featured a 5 mW laser diode with a wavelength of 750 nm. The samples were loaded into a plastic cylinder to achieve an obscuration value between 4% and 8%. Data were collected and analyzed using the LS 13 320 XR ADAPT software version 1.2.323 (Beckman Coulter, FL, USA).

#### 2.3.2. Scanning Electron Microscopy (SEM)

Morphological analysis of the BCS was performed using a JEOL JSM 6060 LV scanning electron microscope (JEOL Ltd., Tokyo, Japan). Microscopic analysis was performed at 100×, 500×, and 1000× magnification with an accelerating voltage of 10.0 kV and a working distance of 14 mm. Before analysis, each sample was mounted on a copper slide with carbon tape.

#### 2.3.3. Fourier Transform Infrared Spectroscopy with Attenuated Total Reflection (FTIR-ATR)

The FTIR spectrum of the BCS was obtained using a Frontier SP8000 infrared spectrophotometer (Perkin Elmer, Waltham, MA, USA) equipped with an ATR accessory (DuraSamplIR II, Smiths Detection, Warrington, UK), over a scan range of 450–4000 cm^−1^, with an average of 32 scans and a resolution of 4 cm^−1^. A background spectrum was collected before the analysis of BCS, and baseline and ATR corrections were applied to the spectrum for further analysis.

#### 2.3.4. Point of Zero Charge (pHpzc)

The zero charge point was determined following a previously described methodology with slight modifications [14]. Briefly, 10 mg of BCS were placed in three tubes, and 10 mL of deionized water adjusted to different pH values (1, 3, 5, 7, 9, and 11, initial pH) were added. The tubes were then vortexed for 10 min, and the final pH of the supernatants was measured using a Mettler Toledo SevenMulti potentiometer (Mettler Toledo, Schwerzenbach, Switzerland) equipped with a combined glass electrode. The pHpzc was calculated by plotting the pH difference (ΔpH: pH_final_ − pH_initial_) against the initial pH, and the point where the line crosses the x-axis represents the pHpzc of the biochar.

#### 2.3.5. Zeta Potential

The electrokinetic potential of BCS was determined by laser Doppler velocimetry (LDV), that is, by electrophoretic mobility, using a Zetasizer instrument (ZetaSizer Pro, Malvern Instruments, Worcestershire, UK) following the recommendations of Ramales-Valderrama et al. [15]. The determinations were performed in triplicate at different pH values (1, 3, 5, 7, 9, and 11) at a temperature of 25 °C with an equilibration period of 120 s, and each measurement comprised 11 cycles to obtain a stable reading. The results were analyzed using ZS Xplorer software v4.0.0 (Malvern Panalytical Ltd., Malvern, UK).

### 2.4. Preparation of the AFB1 Stock Solution

A stock solution of AFB1 (purity ≥98%, Cayman Chemical Company, Ann Arbor, MI, USA) was prepared by dissolving the entire powder from one vial (1 mg) in DMSO and bringing it to a final volume of 20 mL to obtain a concentration of 49,000 ng/mL. This stock solution was subsequently used to prepare the 250 ng/mL (ppb) standard solutions in pH 5.0, 1.2, and 6.8 buffers used in the different experimental assays and the different standard solutions for the calibration curve.

### 2.5. Development of the Analytical Method by Ultra-Performance Liquid Chromatography (UPLC)

AFB1 quantification was performed using an Acquity H-Class ultra-high-performance liquid chromatography (UPLC^®^) system, equipped with a quaternary pump system, an autosampler, and a photodiode array detector (PDA, Waters, Milford, MA, USA). For chromatographic analysis, an Acquity UPLC^®^ HSS T3 column (100 mm × 2.1 mm, 1.8 μm, Waters Corporation, Milford, MA, USA) was used at a temperature of 40 °C. The mobile phase consisted of a mixture of water, MeOH, and ACN (55:25:20) at an isocratic flow of 0.3 mL/min. The sample injection volume was 20 μL, with a total analysis time of 5 min. Detection was performed at a wavelength of 360 nm, and data acquisition and processing were performed using Empower 3 software (Waters, 2010, Milford, MA, USA). The analytical method proved to be accurate and linear in the range of 10 to 250 ng/mL with a limit of detection (LOD) of 2.10 ng/mL and a limit of quantification (LOQ) of 6.38 ng/mL.

#### Filter Selection

Filter selection was evaluated by comparing the AFB1 concentrations obtained after the analysis of six independent unfiltered and filtered solutions through 0.2 μm nylon Acrodiscs (Gelman Sciences Inc., Ann Arbor, MI, USA), regenerated cellulose (CR), cellulose acetate (CA), polyvinylidene fluoride (PVDF), hydrophilic polypropylene (GHP), and polytetrafluoroethylene (PTFE) filters. The standard solution used had an AFB1 concentration of 250 ng/mL at a pH of 6.8.

### 2.6. In Vitro Adsorption Studies

#### 2.6.1. Adsorption Studies of AFB1 in Buffer Solutions

In these adsorption studies, BCS was evaluated at a concentration of 0.05% (*w*/*v*) in AFB1 solutions prepared at pH 5.0 (acetate buffer), 1.2 (chloride buffer), and 6.8 (phosphate buffer), which are the pH conditions of the three main compartments of the avian gastrointestinal tract: crop, proventriculus, and an intestinal section, respectively. These AFB1 solutions were prepared by placing 25 μL of the stock solution and bringing it to a volume of 50 mL with pH 5.0, 1.2, and 6.8 buffer solutions, thus reaching an approximate concentration of 250 ng/mL (ppb) and a DMSO concentration of 0.05%. For this purpose, 2.5 mg of BCS were placed in 15 mL polypropylene Falcon tubes, and 5 mL of AFB1 solutions were added. The tubes were kept in agitation at 19 rpm with the help of an orbital shaker (model 3500, VWR International, Radnor, PA, USA) for 15 min at a temperature of 40 °C in a biochemical oxygen demand incubator (model 2020, VWR, Houston, TX, USA). Subsequently, the samples were centrifuged at 3500 rpm for 10 min at 4 °C (Microfuge R20, Beckman Coulter Life Sciences, Palo Alto, CA, USA), and the supernatants were passed through 0.2 μm Polytetrafluoroethylene filters (PTFE Acrodysc; Gelman Sciences, Ann Arbor, MI, USA) for further UPLC analysis. The studies were performed in sextuplicate, and the buffer solution with AFB1 was considered a positive control, and the buffer solution without AFB1 but with BCS was considered a negative control.

#### 2.6.2. Evaluation of BCS After AFB1 Adsorption by FTIR-ATR

Following the in vitro adsorption studies of AFB1 in buffer solutions, a complementary analysis was performed with the purpose of elucidating the possible mechanism of AFB1 adsorption by BCS. Briefly, the supernatant from the tubes centrifuged in step 2.6.1 was carefully decanted until only the BCS remained at the bottom of the tubes. The tubes were then placed in an oven and maintained at 60 °C for 48 h to ensure complete drying of the BCS. After the drying process was completed, the BCS with and without AFB1 were analyzed by FTIR under the same conditions described in Section 2.3.5, and the spectra obtained were compared to better explain the adsorption mechanism, considering the intensity, shape, or displacement of the characteristic bands of the functional groups.

#### 2.6.3. Adsorption Studies of AFB1 Using an In Vitro Avian Digestion Model

The adsorption of AFB1 by BCS was evaluated in an in vitro model that simulates the physiological conditions of the avian gastrointestinal tract in terms of pH, enzymatic activity, presence of feed, and retention time, considering previous publications with slight modifications [16,17]. Furthermore, two controls were included: a non-commercial zeolite from Taxco, Guerrero, Mexico (size < 250 μm), and a commercial *Saccharomyces cerevisiae* yeast cell wall (SafMannan, Lesaffre Iberica S.A., Valladolid, Spain). For these studies, a biochemical oxygen demand incubator (model 2020, VWR, Houston, TX, USA) was used, set to a temperature of 40 °C and equipped with an orbital shaker operating at 19 rpm (VWR, Houston, TX, USA). The 50 mL polypropylene Falcon tubes used in the model, containing corn (as feed), enzyme solutions, AFB1, and BCS, were held at a 30° angle of inclination to facilitate homogenization.

To simulate the crop, the first compartment of the simulated gastrointestinal tract, 125 mg of ground corn, which was previously passed through a No. 60 mesh to homogenize the particle size, as well as 4.5 mg of BCS (0.05%) and 46 µL of the AFB1 stock solution with a concentration of 49 ppm (µg/mL) were placed in a 50 mL Falcon tube, to achieve a concentration of 250 ng/mL and a DMSO concentration of 0.05% at the end of the model (intestinal section). Subsequently, 5 mL of 0.002 M HCl was added, reaching a pH between 5.0 and 5.2. Thereafter, the tubes were vigorously shaken using a vortex mixer and incubated for 30 min with continuous shaking. Once the first compartment was completed, 3000 U of pepsin were added for each g of feed contained in 1.25 mL of 0.130 M HCl to simulate proventriculus conditions and thus achieve a pH between 1.4 and 2.0. The tubes were shaken again and incubated for another 45 min under constant shaking. Finally, to simulate the intestinal section (pH 6.4–6.8), 6.75 mg of 8 × pancreatin per g of feed was added to 3.25 mL of a 0.1 M sodium bicarbonate solution in each tube and incubated and shaken for a further 2 h.

After the model was completed (3 h 15 min), the tubes were centrifuged at 3500 rpm for 10 min at 4 °C (Microfuge R20, Beckman Coulter Life Sciences, Palo Alto, CA, USA), and an aliquot of the supernatant was collected and filtered through a 0.2 μm PTFE filter. The filtrate was placed in vials to determine the AFB1 concentration by UPLC. The determinations were performed in sextuplicate, including a positive control (without BCS) to calculate the percentage of AFB1 adsorbed under the simulated conditions. The percentage of AFB1 adsorbed was calculated using the following equation:

$$dsorption\left(\%\right)=\frac{\left(C_{i}-C_{s}\right)}{C_{i}}\times 100$$

where *C_i_* is the concentration of AFB1 in the positive control samples (ng/mL), and *C_s_* is the concentration of AFB1 in the samples with adsorbents (ng/mL).

### 2.7. Statistical Analysis

Data derived from filter selection, as well as from BCS adsorption studies of AFB1 in buffer solutions and in the in vitro avian model, are presented as mean ± standard error (SE). After verifying data normality and homogeneity of variance using the Shapiro–Wilk and Levene tests, respectively, differences between experimental groups were assessed using a one-way analysis of variance (ANOVA) followed by a Tukey post hoc test (*p* < 0.05) using JMP^®^ Student Edition, version 19.0.3 (JMP Statistical Discovery LLC, Cary, NC, USA) and GraphPad Prism version 10.4.2 (GraphPad Software, San Diego, CA, USA).

## 3. Results and Discussion

### 3.1. Characterization of the Biochar

#### 3.1.1. Particle Size Analysis

In this study, a biochar derived from coconut shells (BCS) was obtained with a yield of 24.0%, with a particle size distribution that was not entirely homogeneous, and an average size of 69.45 ± 35.78 µm (Table 1). These results are consistent with other studies in which the yield of a biochar derived from young coconut shells was 25.7% at 400 °C [18] and the particle size was similar (50 µm and 50–250 µm) under the same production conditions, but the milling process has a significant impact, as particle sizes as small as 18 µm can be achieved with ball mills [19,20]. However, it is known that temperature plays an important role since, as it increases, particle size and yield decrease due to the decomposition of hemicellulose, cellulose, and a fraction of lignin, as well as volatile compounds [20,21]. Although particle size depends on the composition of the agro-industrial waste and the pyrolysis temperature to obtain the corresponding biochar, it has been reported that this has a minimal influence on properties such as adsorption since its elemental composition, lignocellulosic residues, and pore size are more important when these materials are obtained at temperatures between 300 and 400 °C [22,23].

#### 3.1.2. Morphological Analysis by SEM

**Figure 1:**
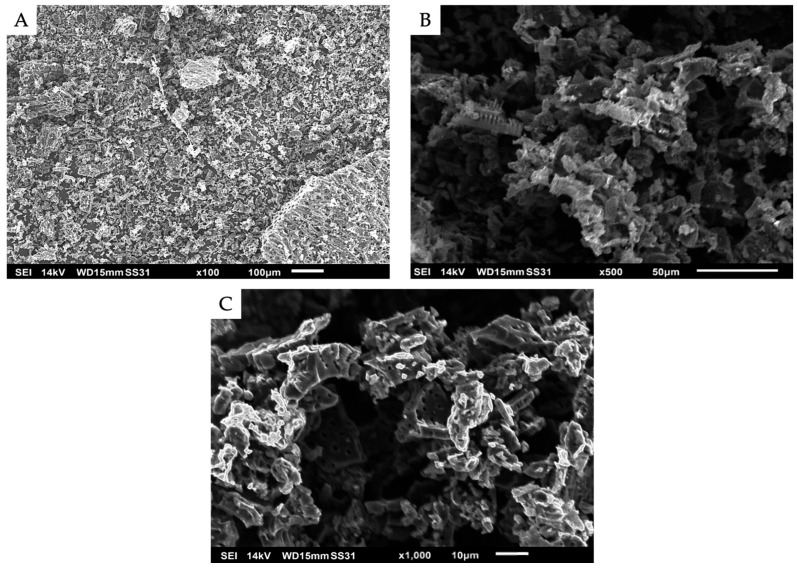
Micrographs of biochar derived from agro-industrial waste from coconut shell (BCS) obtained at 100× (**A**), 500× (**B**), and 1000× (**C**).

The surface morphology and microstructure of BCS were analyzed using SEM (Figure 1). At low magnifications (100× and 500×), heterogeneous structures with fractured edges and an irregular, rough surface were observed, while at high magnification (1000×), microchannels and open pores with circular and elongated morphologies were evident, with estimated diameters between 0.5 and 1 µm (macropores). These cavities are associated with the natural fibrous structure of the lignocellulosic cell wall, and previous studies of coconut shell-derived materials have reported similar morphologies after activation [24]. Furthermore, it has been suggested that these heterogeneous microstructures favor the adsorption of contaminants such as mycotoxins [25].

#### 3.1.3. FTIR-ATR Analysis

**Figure 2:**
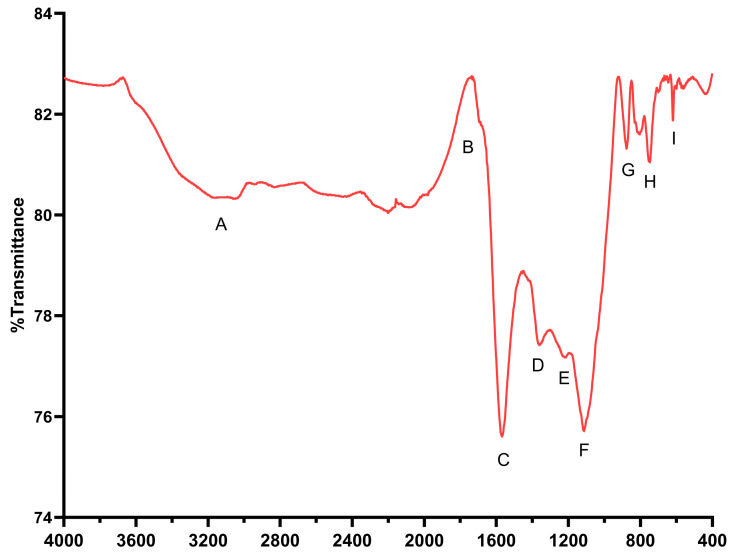
Fourier transform infrared (FTIR) spectrum of biochar derived from agro-industrial coconut shell waste (BCS).

The FTIR-ATR spectrum of the BCS obtained in the spectral range of 4000–400 cm^−1^ is shown in Figure 2 The bands corresponding to the stretching vibrations of the functional groups present in the BCS are listed in Table 2. The IR spectrum of the BCS showed nine significant bands. The broad band of moderate intensity found between 3500 and 3300 cm^−1^ corresponds to the O–H stretching of the hydroxyl groups, which is characteristic of cellulose residues, but it has been described that as the carbonization temperature increases, this band is reduced due to the formation of condensed carbon structures [20]. At 1700 cm^−1^, a low-intensity band was observed that is associated with aromatic and olefinic C=C stretching vibrations and is produced during the carbonization stage [20]. This band suggests the presence of organic compounds, such as ketones, lactones, aldehydes, and carboxylic acid groups, which are common functional groups in materials derived from natural sources [26]. Aromatic C–O stretching vibrations in the lignin ring were observed at 1571 cm^−1^. The intensity of this band is related to a higher aromaticity of the biochar due to the carbonization temperature, meaning that the higher the temperature, the greater the aromaticity [20,26]. A representative band corresponding to C=C stretching aromatic vibrations was observed at 1354 cm^−1^, which can be intensified as the carbonization temperature increases [20]. Furthermore, the C–O–C stretching vibration band was found at 1212 cm^−1^ [27]. Likewise, a band was observed at 1111 cm^−1^, which corresponds to the C–O stretching vibrations of the alkyl/aryl ether functional groups. However, it is known that this band decreases as the carbonization temperature increases due to the condensation of aromatic carbons [20]. The band observed at 875 cm^−1^ has been reported to be characteristic of a strong aromatic band of the C–H phenyl ring [20]. Finally, the bands found between 750 and 500 cm^−1^ were characteristic of the stretching vibrations of the aromatic C–H and C–C of the biochar skeleton [26].

In this context, the functional groups attributed to O–H, C=O, and C–O stretching vibrations, as well as the greater amount of aromatic compounds in the BCS, play an important role in the adsorption of contaminants such as AFB1 [28].

#### 3.1.4. Point of Zero Charge (pHpzc) and Zeta Potential

**Figure 3:**
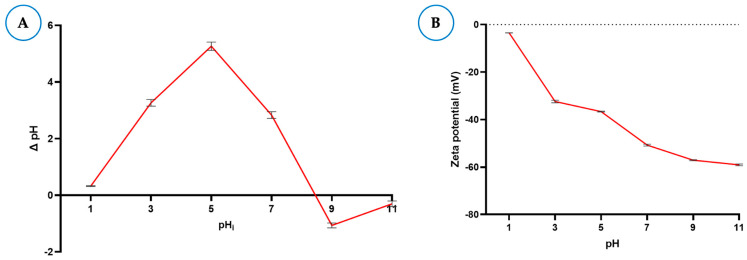
(**A**) Point of zero charge (pHpzc) and (**B**) Zeta potential of biochar derived from agro-industrial coconut shell waste (BCS) at different pH values.

The point of zero charge (pHpzc) of the BCS was determined using the pH variation method, where ΔpH was plotted as a function of the initial pH (Figure 3A), and its importance lies in the fact that the surface properties of the biochar can be known, which are important for the adsorption phenomena of contaminants [29,30]. The pHpzc value of the BCS was 8.4, which means that at this pH, the positive and negative charges are in equilibrium. These results are consistent with another study that reported a pHpzc value of 8.3 for biochars derived from coconut fiber [31]. Furthermore, this analysis confirmed that at pH levels less than 8.4, BCS is positively charged due to the protonation of the functional groups, while at higher pH levels, it predominantly acquires a negative charge given the deprotonation of the hydroxyl groups [32]. However, electrostatic interactions do not play a decisive role in the adsorption of AFB1 since it is not charged across the entire pH range.

In fact, the alkaline pH of biochars is known to be related to lignin, hemicellulose, or cellulose residues and the presence of oxygen-rich functional groups such as γ-pyrone, chromene, diketone, or quinine groups [22]. Furthermore, the alkalinity of biochars is known to depend on the source type, resulting in a higher concentration of salts, such as potassium and calcium chlorides, and basic cations like Mg^2+^, Ca^2+^, and K^+^, as well as on the carbonization temperature, since pH increases with increasing temperature. Therefore, pH values can vary significantly [20,22]. Therefore, pHpzc values can vary significantly [29,31,32].

Figure 3B shows the zeta potential of BCS at different pH values. This determination is important for understanding the surface characteristics with respect to charge [33]. The zeta potential of BCS was negative across the different pH values evaluated, starting with a value of −3.6 mV at pH 1 and followed by values of −32.9, −36.4, −50.0, −56.8, and −58.5 mV for pH 3, 5, 7, 9, and 11, respectively. Interestingly, the BCS did not exhibit isoelectric points. These results suggest that the surface charge of BCS is negative and that it also contains a large number of functional groups derived from the very negative zeta potential [34]. Although there are no studies that report the zeta potential of biochars derived from coconut shell with respect to pH, some studies have reported zeta potentials around −42.0 mV at pHs between 6.7 and 8.7 [34,35]. In addition to the fact that the zeta potential is related to the surface charge, it is also indicative of greater colloidal stability and adsorption capacity derived from the ionic interactions that can occur between hydroxide ions (OH−) and other anions on the surface of the biochar and the functional groups that are part of the contaminants [36,37].

### 3.2. Analytical Method by Ultra-Performance Liquid Chromatography (UPLC)

**Figure 4:**
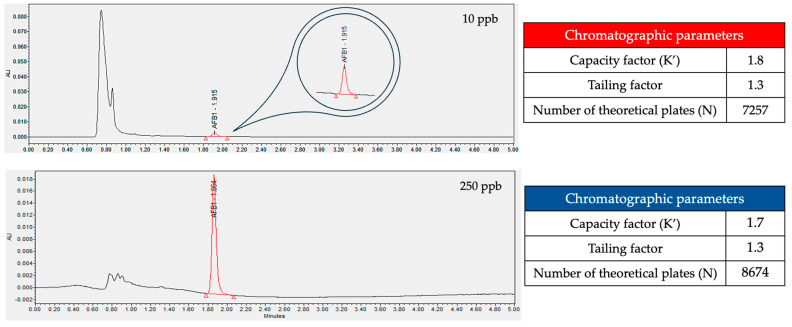
Chromatogram of AFB1 at 10 and 250 ng/mL with their respective chromatographic parameters.

A UPLC–PDA analytical method was developed for the quantification of AFB1, which proved to be linear, accurate, and specific in a concentration range of 10 to 250 ng/mL of AFB1. Figure 4 shows the chromatograms obtained for standard solutions of AFB1 at concentrations of 10 and 250 ng/mL, with a retention time of approximately 1.9 min. The system was considered suitable for the analysis of AFB1 since a capacity factor (K’) greater than 1.5, a tailing factor less than 2, and a number of theoretical plates greater than 2000 were observed [38,39]. Furthermore, it showed a limit of detection (LOD) of 2.10 ng/mL and a limit of quantitation (LOQ) of 6.38 ng/mL, according to the signal-to-noise ratio obtained from the chromatograms. There are two groups of methods that allow the detection and quantification of AFB1: chromatographic and bioassays. The latter are the most used, but they present the greatest problem of inconsistency because the antibodies used, which are considered specific, can react with other substances. Therefore, bioassay methods should be used for exploratory purposes, and their results should be compared with those of chromatographic methods for confirmation. The chromatographic methods used for confirmation include those with fluorescence detectors or those coupled to mass spectroscopy [40]. Although the chromatographic methods mentioned above are more sensitive and specific, they are also more expensive compared to UV detectors [41], which limits their use. In this regard, the developed method offers advantages, beginning with the low concentration of AFB1 that can be reliably quantified and the short retention time compared to other reported methods [42].

**Figure 5:**
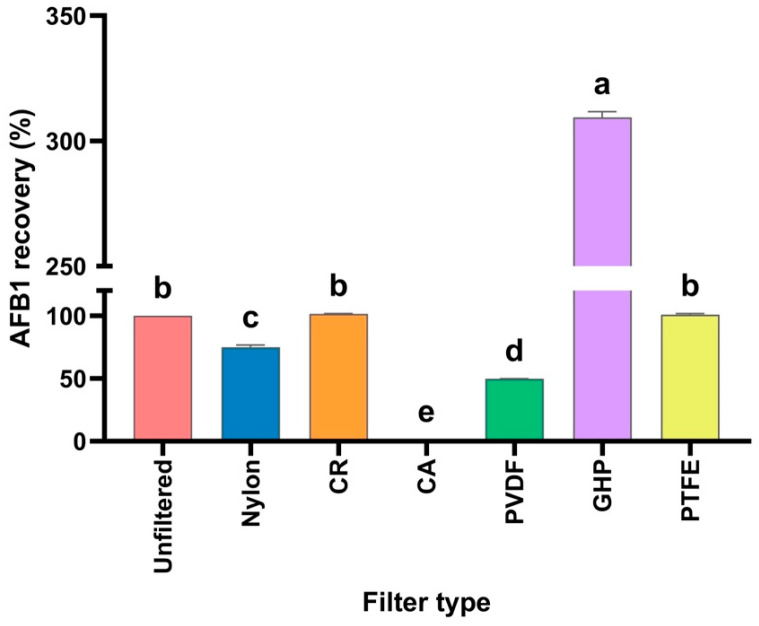
Influence of the filter on the recovery of AFB1 (250 ng/mL). ^a–e^ Bars with different letters are considered significant (*p* < 0.05). The results are given as the mean ± SE (*n* = 6). CR: regenerated cellulose, CA: cellulose acetate, PVDF: polyvinylidene fluoride, GHP: hydrophilic polypropylene, and PTFE: polytetrafluoroethylene.

The development of the analytical method also considered evaluating the influence of the filter, since during the processing of the AFB1-contaminated corn samples in the in vitro avian model, centrifugation and filtration processes were performed. For this purpose, AFB1 solutions at 250 ng/mL filtered through six different types of 0.2 μm Acrodiscs were compared with unfiltered solutions (Figure 5). The results showed that the filtration process had no influence when using CR and PTFE filters, as no significant differences were observed compared to the unfiltered solutions. However, we decided to filter the samples with PTFE because it allowed for achieving solutions free of particles and extractable impurities [43]. In the case of the nylon, PVDF, and CA filters, 24.98%, 50.26%, and 98.64% of AFB1 were retained, respectively. Interestingly, the GHP filter yielded a recovery of 309.44% of AFB1, mainly because the filter can release extractable impurities, which can affect the analysis [43].

### 3.3. Adsorption Studies of AFB1 in Buffer Solutions

**Figure 6:**
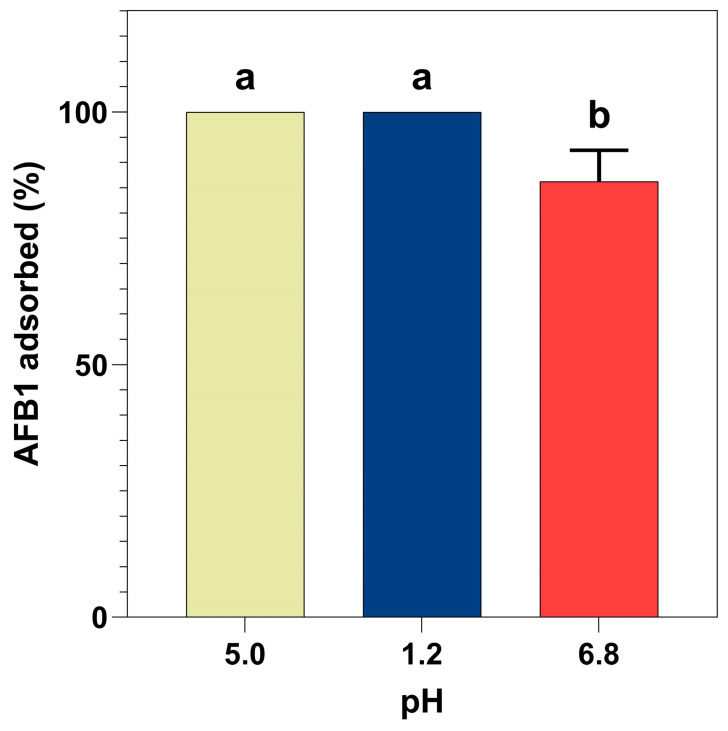
Adsorption capacity of biochar derived from agro-industrial coconut shell waste (BCS) at 0.05% under different pH, considering an AFB1 concentration of 250 ng/mL. ^a,b^ Bars with different letters are considered significant (*p* < 0.05). The results are given as the Mean ± SE (*n* = 3).

Figure 6 shows the adsorption capacity of BCS on AFB1 found in buffer solutions at pH 5.0, 1.2, and 6.8. The pH values evaluated correspond to the averages reported for the three main compartments of the gastrointestinal tract of broiler chickens: crop (5.0), proventriculus (1.2), and an intestinal section (6.8) [44]. The adsorption capacity of BCS on AFB1 (250 ng/mL) at both pH 5.0 and 1.2 was close to 100%, while at pH 6.8, the adsorption of AFB1 was 86.24%. These results are similar to another study in which, at pH 2.0, the AFB1 adsorption capacity of a biochar derived from coconut shell was 98.7% and 93.3% at pH 6.8, but considering an AFB1 concentration of 0.1 and 1.0 ppm (µg/mL) and 0.25% of the biochar, a concentration five times higher than that used in the present study [13]. Another study reported that the adsorption capacity of an activated carbon obtained from coconut shells was 98.5% at pH 6.8, but it was achieved at an AFB1 concentration of 10 ng/mL and an activated carbon concentration of 0.5%, which is 25 times lower and 10 times higher, respectively, compared to the present study [45]. However, it is known that the variation in the adsorption process is due to the composition of the raw material and the process of obtaining these carbonaceous adsorbents [20,22].

#### AFB1 Adsorption Mechanism

**Figure 7:**
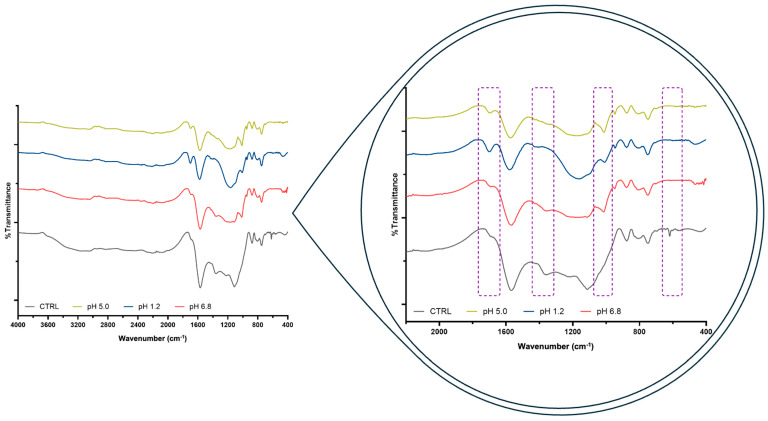
FTIR spectra of the BCS before and after AFB1 adsorption studies in buffer solutions at pH 5.0, 1.2, and 6.8. The dotted purple lines indicate the regions where changes in spectral behavior occur.

The key factors that influence the adsorption of carbonaceous materials are strongly related to the size and volume of the pores (micropores) and the contact surface area, as well as the polarity, solubility, and size of the AFB1 [46,47], with the main adsorption mechanisms being hydrophobic interactions, van der Waals forces, hydrogen bonds, and, in certain cases, electrostatic interactions [47]. In this regard, to better determine the BCS adsorption mechanism, an FTIR analysis was performed on the biochars before and after the adsorption studies in buffer solutions at pH 5.0, 1.2, and 6.8 (Figure 7). The results show changes in spectral behavior, beginning with the appearance of a new band at 1017 cm^−1^ in the BCS used in the AFB1 adsorption studies compared to the control BCS without AFB1 exposure. This band is related to the symmetric stretching vibration of the ether group (=C–O–C) present in the furan ring structure of AFB1 [48,49], which suggests adsorption of AFB1 onto BCS due to dipole–dipole intermolecular interactions between the ether groups (=C–O–C) of AFB1 and the C–O groups present on the surface of BCS [50]. Furthermore, the band corresponding to aromatic and olefinic C=C stretching vibrations (1700 cm^−1^) increased in intensity in the BCS exposed to AFB1 compared to the control BCS. This behavior is due to π–π stacking interactions between the aromatic rings of AFB1 and the aromatic C=C groups of the BCS [51], resulting in increased AFB1 adsorption. However, pH plays an important role since this band has lower intensity at pH 6.8, which could have caused less adsorption of AFB1. In fact, it has been reported that π–π stacking interactions decrease as pH increases due to electrostatic repulsive forces [52], which explains the observed behavior. In contrast, the bands found at 1354 cm^−1^ and 619 cm^−1^, which correspond to aromatic C=C stretching vibrations and carbonaceous C–C skeleton in the control BCS, respectively, disappeared in the BCS exposed to AFB1 at different pH values, which is related to hydrophobic interactions [53].

### 3.4. Adsorption Studies of AFB1 Using an In Vitro Avian Digestion Model

Currently, in vitro models used to evaluate the adsorption capacity of AFB1 adsorbent materials are mainly based on the use of buffer solutions that simulate pH, leaving aside the effect of feed, enzymatic activity, and residence times in the different compartments of the gastrointestinal tract [54]. Therefore, this can lead to an overestimation of results, which could result in ineffective treatments or unexpected results in animals due to interactions that can compromise the adsorption capacity of AFB1 by the adsorbent materials, since corn, the main component of poultry feed, has been reported to have adsorbent capacity given its composition (cellulose, xylan, lignin, and arabinoxylan) [55].

**Figure 8:**
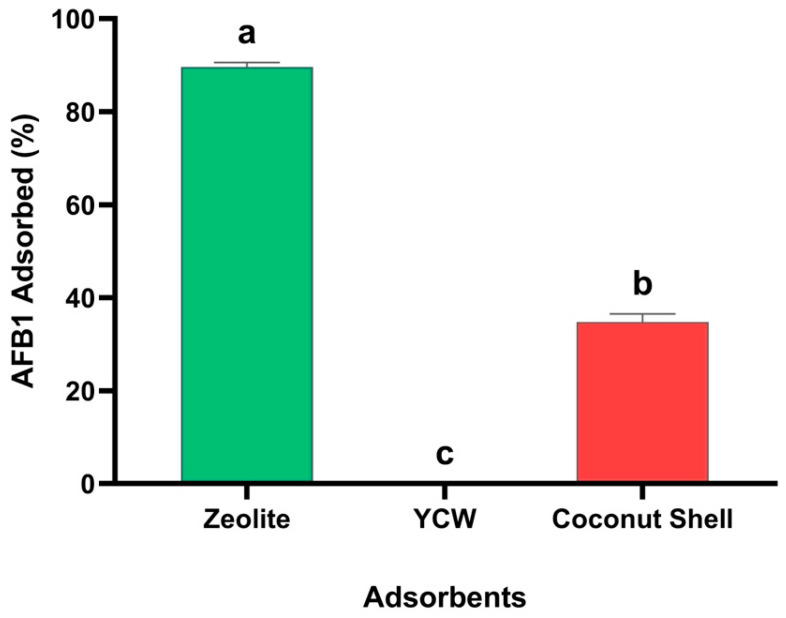
The adsorption capacity of biochar derived from agro-industrial coconut shell waste (BCS) at 0.05% evaluated in the in vitro avian digestion model, considering an AFB1 concentration of 250 ng/mL. ^a–c^ Bars with different letters are considered significant (*p* < 0.05). Results are expressed as mean ± SE (*n* = 6). YCW: Yeast Cell Wall.

Considering this context, a previously reported in vitro digestion model was used with minor modifications [16,17], which simulates the conditions of the gastrointestinal tract of birds in terms of the presence of feed, pH, enzymatic activity, and residence time in the crop (pH 5.0), proventriculus (pH 1.2 with pepsin), and an intestinal section (pH 6.8 with pancreatin). The results show that the AFB1 adsorption capacity of BCS was 32.96% in the last simulated compartment of the in vitro model (intestinal section, pH of 6.8) (Figure 8). These adsorption results are 53.28% lower compared to the in vitro model that only used pH 6.8 buffer solutions (86.24%), thus indicating the importance of considering factors that can affect the adsorption capacity of materials. In fact, to compare the results obtained with the BCS, the AFB1 adsorption capacity of a commercial yeast cell wall (YCW)-based product was evaluated, revealing zero adsorption capacity. In contrast, the non-commercial zeolite showed an AFB1 adsorption of 89.64%. The reduction in BCS adsorption capacity can be explained by the occupation or physical obstruction of BCS active sites, as well as the blocking of micropores due to their size by the maize components used in the model (non-digestible fiber, lignin, cellulose), resulting in a reduction in AFB1 adsorption [56]. Unlike BCS, zeolite showed better adsorption capacity because the pore dimensions are known to be close to molecular sizes, resulting in better adsorption [57]. However, these types of adsorbents are nonspecific and may compromise feed efficiency in broiler chickens due to factors such as the slowing of feed transit through the intestine, the immobilization of enzymes, and the modulation of the intestinal microbiota [58]. Therefore, BCS can be positioned as a safer, more sustainable alternative with a low risk of nutrient binding [13,59]. In the case of YCW, its AFB1 adsorption efficiency has been reported to be variable (20.46 to 54.48%) and pH-dependent due to the β-glucans, being more effective at acidic pH [60]. Furthermore, it also exhibits fewer binding sites compared to biochars and zeolites, so research has focused on improving cell wall components to increase binding sites and thus improve its adsorption efficiency [60]. These phenomena, combined with the interactions of the corn components, resulted in a complete blockage of its adsorption in the model.

## 4. Conclusions

A biochar derived from agro-industrial coconut shell waste (BCS) with good AFB1 adsorbent properties was developed, as well as an analytical method by UPLC-PDA that proved to be linear, accurate, and specific for the quantification of AFB1. The adsorbent capacity of BCS at 0.05% was different in the two in vitro models used. While in the in vitro model that only considered buffer solutions at pH 5.0, 1.2, and 6.8, the adsorption of AFB1 was higher, in the in vitro model that simulates the gastrointestinal conditions of the broiler chicken, it was 53.28% lower due to the interactions that can occur between the corn used as feed and the biochar, which is the closest to real conditions, so the results would be more representative and not overestimated. The mechanisms by which these biochars carry out their adsorption capacity include the size and volume of the pores (micropores), hydrophobic interactions, van der Waals forces, hydrogen bonds, and π–π stacking interactions. Therefore, the inclusion of BCS in feed could be a viable alternative to mitigate the problems related to AFB1 in poultry production. However, chemical modifications of BCS through its activation with alkalis or acidic agents could be a viable strategy to improve its adsorption properties derived from the increase in surface area and pore volume of BCS, as well as the generation of functional groups in its structure. Currently, studies are being conducted in broiler chickens to validate the results and determine if the in vitro model is optimal to ensure the effectiveness of adsorbents before conducting studies in animals.

## Figures and Tables

**Table 1:** Particle size of the biochar derived from coconut shells (BCS) determined by laser diffraction (DLS).

Biochar	Mean ± SD (µm)	Median (µm)	D_10_ (µm)	D_90_ (µm)
BCS	69.45 ± 35.78	67.03	23.29	118.90

SD: standard deviation.

**Table 2:** Assignment of the bands and functional groups present in the biochar derived from agro-industrial coconut shell waste (BCS).

Band	Wavenumber (cm^−1^)	Functional Group
A	3500–3300	O–H stretching vibrations.
B	1700	Aromatic and olefinic C=C stretching vibrations.
C	1571	Aromatic C–O stretching vibrations.
D	1354	Aromatic C=C stretching vibrations.
E	1212	C–O–C stretching vibrations.
F	1111	C–O stretching vibrations.
G	872	C–H stretching vibrations of the phenyl ring.
H and I	750–500	Aromatic C–H stretching vibrations and carbonaceous C–C skeleton.

## Data Availability

The databases used and analyzed during the current study are available from the corresponding author.

## Abbreviations

The following abbreviations are used in this manuscript:

AFB1	Aflatoxin B1
BCS	Biochar Derived from Agro-industrial Coconut Shell Waste
CA	Cellulose Acetate
CR	Regenerated Cellulose
FTIR-ATR	Fourier Transform Infrared Spectroscopy with Attenuated Total Reflection
GHP	Hydrophilic Polypropylene
pHpzc	Point of Zero Charge
PVDF	Polyvinylidene Fluoride
PTFE	Polytetrafluoroethylene
SEM	Scanning Electron Microscopy
UPLC	Ultra-Performance Liquid Chromatography
